# The Comprehensive Analyses of Genomic Variations and Assessment of TMB and PD-L1 Expression in Chinese Lung Adenosquamous Carcinoma

**DOI:** 10.3389/fgene.2020.609405

**Published:** 2021-02-17

**Authors:** Yong Cheng, Yanxiang Zhang, Yuwei Yuan, Jiao Wang, Ke Liu, Bin Yu, Li Xie, Chao Ou-Yang, Lin Wu, Xiaoqun Ye

**Affiliations:** ^1^Department of Respiratory Diseases, The Second Affiliated Hospital of Nanchang University, Nanchang, China; ^2^Berry Oncology Corporation, Beijing, China

**Keywords:** adenosquamous carcinoma, EGFR, lung, next generation sequencing, PD-L1, somatic variations, TMB

## Abstract

The poor prognosis and fewer treatment option is a current clinical challenge for patients with lung adenosquamous carcinoma (ASC). The previous studies reported that tumor mutational burden (TMB, numbers of mutation per Megabase) is a predictor of clinical response in trials of multiple cancer types, while fewer studies assessed the relationship between TMB level and clinical features and outcomes of lung ASC. Herein, the present study enrolled Chinese patients with lung ASC. DNA was extracted from formalin-fixed paraffin-embedded tumor samples and subjected to next generation sequencing (NGS), and the 457 cancer related genes were evaluated. The results demonstrated that 95 unique genes with somatic variations were identified in the enrolled patients. The top three of high frequency gene mutations were *TP53, EGFR, PIK3CA* with rates of 62% (13 cases), 48% (10 cases), and 14% (3 cases), respectively. We identified TMB value was significantly correlated with pathological stages (*p* < 0.05) and invasion of lymph node (*p* < 0.05). However, TMB value was not significantly correlated to other clinicopathologic indexes, for examples, age, sex, smoking history, tumor size, as well as *TP53* and *EGFR* mutations in lung ASC. Moreover, TMB value was associated with the overall survival (*p* < 0.01), but not with the relapse-free survival (*p* = 0.23). In conclusion, this study indicated that lung ASC with high TMB might be associated with the invasion of lymph node and short overall survival. Immunotherapy might be a promising treatment option for lung ASC patients with high TMB.

## Introduction

Worldwide, lung cancer is the most prevalent cause of cancer related death (Siegel et al., 2018). Adenosquamous carcinoma (ASC) is a small subtype of non-small-cell lung cancer (NSCLC), accounting for <4% of all patients with NSCLC (Uramoto et al., 2010; Li and Lu, 2018). It is defined that lung ASC is a mixed-type tumor, comprised of adenocarcinoma and squamous cell carcinoma. Each component accounts for at least 10% of the total tumor cells, according to the tumor classification by the fifth edition of world health organization (WHO) (Travis et al., 2015). It is reported that lung ASC displays the worse prognosis than other types of NSCLC. Lung ASC is resistant to the treatment of adjuvant chemotherapy, and more probably to occur local recurrence or distant metastasis in comparison with other histologic types of NSCLC (Hsia et al., 1999; Nakagawa et al., 2003; Maeda et al., 2012).

In recent years, the important advancements have been achieved in NSCLC treatments (Herbst et al., 2018; Testa et al., 2018). For example, the small molecule tyrosine kinase inhibitors (TKIs) were effective for patients with advanced lung adenocarcinoma with the somatic mutation of *epidermal growth factor receptor* (*EGFR*) and the rearrangement of *echinoderm microtubule-associated protein-like 4* (*EML4*) with *anaplastic lymphoma kinase* (*ALK*) (Paez et al., 2004; Soda et al., 2007; Robichaux et al., 2018; Ramalingam et al., 2020). Interestingly, a few case reports and retrospective studies have demonstrated that EGFR-TKIs therapies were effective for the selected patients with advanced ASC of the lung (Song et al., 2013; Kurishima et al., 2014; Fan et al., 2017; Zhang et al., 2018; Lin et al., 2020). Therefore, besides of *EGFR* mutation, the continued research is required to identify more cancer-related gene mutations and the corresponding targeted agents or combined therapies to improve outcomes for lung ASC.

Immune checkpoint inhibitor (ICI) therapies have shown significant benefit in treatment of patients with NSCLC (Herbst et al., 2018), for example, pembrolizumab treatment achieved better clinical outcomes compared to platinum-based chemotherapy in advanced NSCLC patients with high expression of programmed death ligand 1 (PD-L1) in tumor cells (Herbst et al., 2016; Reck et al., 2016). Besides of programmed death 1 (PD-1) and its ligand PD-L1, the resent studies indicated that tumor mutational burden (TMB) could predict clinical outcomes in multiple cancer types, including lung cancer patients receiving immunotherapy (Rizvi et al., 2015, 2018; Samstein et al., 2019). However, there is still lack of prospective data and retrospective study to comprehensively depict the genomic landscape and immune biomarkers, as well as their association with the clinicopathologic features in patients with lung ASC.

To address the limited knowledge, we performed this study in patients with surgically resected lung ASC to evaluate (1) the genomic variations and its correlation with TMB and PD-L1 expression and (2) the clinical relevance of TMB and PD-L1 expression, including clinicopathologic features, relapse-free survival (RFS), and overall survival (OS). Meanwhile, we compared the data of lung ASC to other ethnicities as well as other subtypes such as adenocarcinoma and squamous cell carcinoma.

## Materials and Methods

### Patients and Samples

All the enrolled patients with lung adenosquamous carcinoma (ASC) underwent surgical resection from the Second Affiliated Hospital of Nanchang University between April, 2014 and May, 2019. The criteria of the enrolled patients were as follows: (1) pathological diagnosis of lung ASC according to the tumor classification in the fifth edition of WHO, each component of adenocarcinoma and squamous cell carcinoma at least 10% of the tumor cells; (2) patients without anticancer treatment before surgery; (3) availability of complete medical records, including patient's age, gender, smoking history, immunohistochemistry results, pathological reports, operation time and surgical approach, medication records, tumor response assessment. All the enrolled patients accepted and signed the informed consent, the protocol was approved by the Ethics Committee of medical research, the Second Affiliated Hospital of Nanchang University.

### FFPE Preparation and Genomic DNA Extraction

After surgical resection, tumor tissues and normal tissues (incision margin 5 cm away from the tumor) were fixed with formalin, subsequently embedded in paraffin (FFPE). Genomic DNA was extracted from each FFPE sample using the GeneRead DNA FFPE Kit (Qiagen, USA) according to the manufacturer's protocol, respectively.

### NGS Based Large-Gene Panel Test

To construct the pre-library, genomic DNA was digested into ~200 bp fragments by enzymatic method, then subjected to end repairing, A-tailing, adapter ligation and universal amplification. Purified pre-library was hybridized with a customized biotin probe pool (the 457 genes panel, Berry Oncology, Peking, China) to capture target fragments (Supplementary Table 1). Captured fragments were amplified with universal primers and purified to acquire the final library. The library of paired-end multiplex samples were sequenced with the NovaSeq 6000 System. Sequencing depth was ~2,000 x per sample.

The generated sequences were trimmed, low-quality-filtered, and subjected for variant calling. Variants was filtered for nonsynonymous SNPs, indels and spliced variants. Somatic variations were identified with variant allele frequency (cutoff ≥ 3%) and cancer hotspots were screened with variant allele frequency (cutoff ≥ 1%) and at least 20 high-quality reads.

The tumor mutation burden (TMB) was determined by the number of all the nonsynonymous mutation and indel variants per magabase of coding regions. The 457 gene panels cover the coding region of 1,141,951 bp. Hence, TMB was calculated with the number of all the nonsynonymous mutations and indel variants/1.14 Mb. The threshold of high TMB was set to 10 according to the previous studies (Hellmann et al., 2018; Barroso-Sousa et al., 2020).

### Immunohistochemistry Assays

Immunohistochemistry assays were performed on FFPE sections using a primary anti-PD-L1 (SP263) rabbit monoclonal antibody (Roche) and a secondary anti-rabbit-IgG antibody (ZSGB-BIO, Beijing, China), then detected with DBA detection kit (ZSGB-BIO, Beijing, China). PD-L1-positive was determined if membrane staining exhibited in ≥ 25% of tumor cells in the tumor sample, as described in the previous study (Shi et al., 2017).

### Statistics

R Foundation for Statistics Computing, R script (v3.6.0) was used to perform the statistics analysis. Fisher's exact test was used to analyze the relationship between TMB and clinical indexes. Kaplane-Meier method was used to estimate progress-free survival and overall survival. *p* < 0.05 was defined as statistically significant.

## Results

### Clinicopathological Characteristics of the Enrolled Patients

In total, 21 patients with lung adenosquamous carcinoma (ASC) were finally enrolled in this study. The median age at diagnosis was 63 years (range: 49–75), eleven were male and ten were female, seven were smokers and 14 were non-smokers. The tumors were stage I, II, and III in nine (42.9%), three (14.3%), and nine (42.9%) cases, respectively. Ten patients were with adeno cells accounted for 10%-50% and eleven patients were with adeno cells accounted for 51–90%. The clinical information of patients was overviewed in Table 1. The characteristics of each patient were listed in Supplementary Table 2.

**Table 1:** The overview of clinical information of enrolled patients.

**Patients (*****n*** **=** **21)**	
Age	
Median	63
Range	49-75
Sex (%)	
Male	11 (52.4%)
Female	10 (47.6%)
Smoking history (%)	
Current	7 (33.3%)
Former	0
Never	14 (66.7%)
Pathological stage (%)	
I	9 (42.9%)
II	3 (14.3)
III	9 (42.9%)
Histologic type	
Adeno cells accounted for 10–50%	10
Adeno cells accounted for 51–90%	11

### Somatic Variation Detection

**Figure 1:**
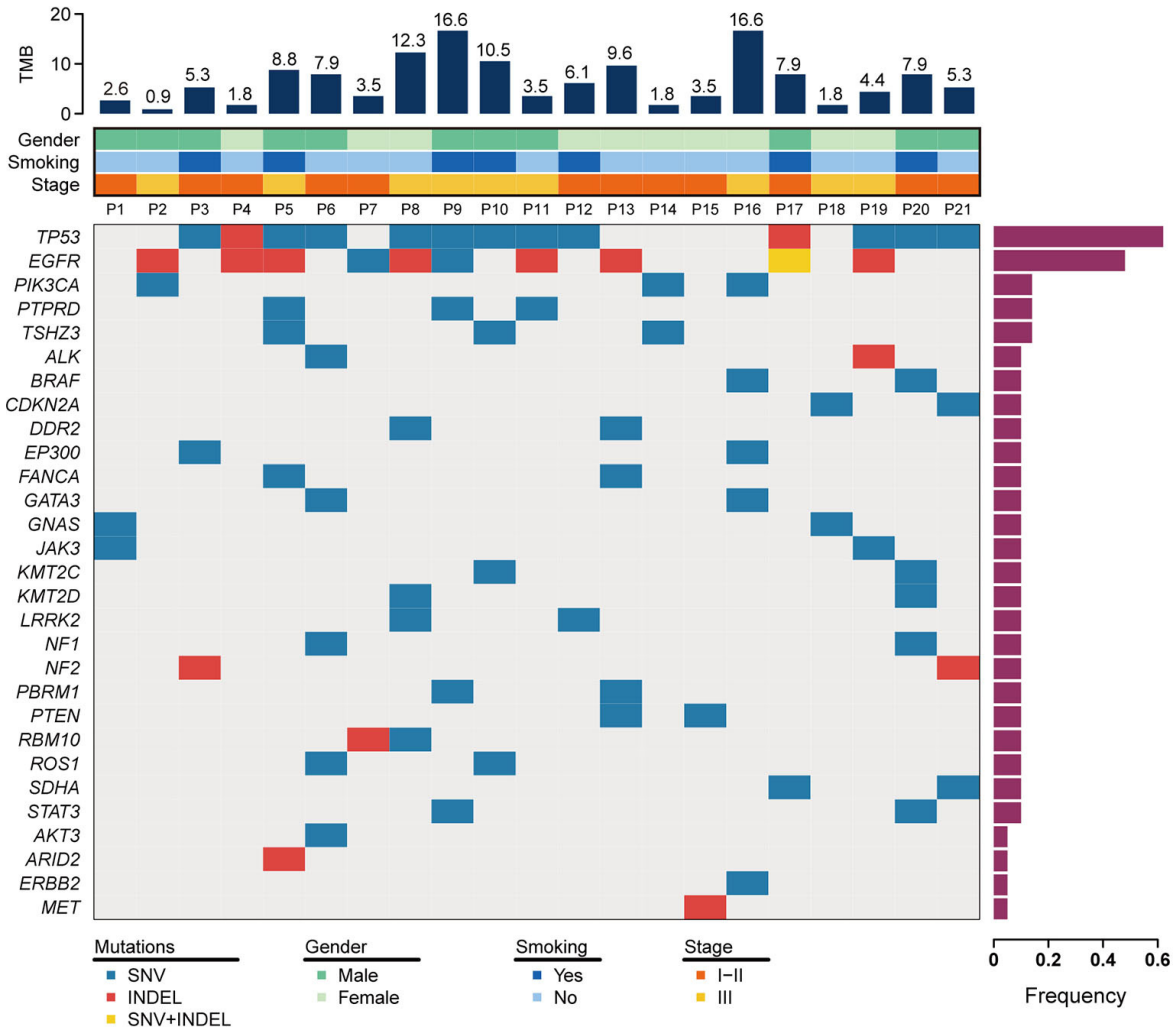
Somatic mutation landscape for 21 patients with lung ASC. The top 30 mutant genes are displayed. The frequency of mutated genes are shown in a histogram on the right. Tumor mutation burden (TMB) and clinical features are illustrated in the upper two panels. SNV+INDEL represents the case carrying both of mutation types of SNV and INDEL.

To discover somatic variation in ASC, DNA was extracted from formalin-fixed paraffin-embedded samples and subjected to NGS based large-gene panel test. The somatic mutations of each tumor sample were analyzed and summarized in Figure 1 and Supplemental Table 3. Our study identified 95 unique genes with somatic variations. Among those, the top three of high frequency gene mutations were *TP53, EGFR, PIK3CA* with rates of 61.9% (13 cases), 47.6% (10 cases), and 14.3% (3 cases), respectively. Coexisting mutations were detected in *TP53* and *EGFR* with rate of 33.3% (7 cases), *EGFR*, and *PIK3CA* with rate of 4.8% (1 case).

Mutations of the *TP53* gene are universal in lung cancer, with mutation rate of about 50% in NSCLC (Mogi and Kuwano, 2011). In lung ASC, our study indicated *TP53* was a highest frequency mutation gene, with a mutation rate of 62%. The common mutation was detected in exon 8 (5 cases), exon 7 (4 cases), exon 6 (2 cases), and exon 4 (1 case). The hotspot mutations of TP53 p.R248Q and TP53 p.R248W were detected in p11 and p19, respectively, which are the target of APR-246 drug. At present, FDA has approved APR-246 in combination therapies to treat myelodysplastic syndromes with TP53 mutation, while more clinical trials are required to prove the efficacy of the drug in treating patients with lung cancer.

For *EGFR* gene, deletion in exon 19 was the most common mutation (7 cases), and the single nucleotide variation in exon 21 resulting in EGFR p.L858R variant was observed only in one case. These mutations are related to the increase of sensitivity to tyrosine kinase inhibitors. The drug resistant mutation was detected in one case harboring insertion mutation in exon 20, while none of T790M variant was observed in this study. Two variants of in-frame deletion in exon 19 and single nucleotide variation resulting in EGFR p.E758D were coexisted in one case, as shown in Figure 1 and Supplemental Table 3.

In addition to *EGFR* mutations, a set of genes involved in the *PI3K* signaling pathway were observed in seven lung ACS cases in our study. Two cases (p2 and p16) harbored a single nucleotide variation in exon 9 of *PIK3CA*, resulting in a p.E545K variant in the helical region, and one case (p14) harbored a mutation in exon 21 of *PIK3CA* which generated a p.H1047L variant in p110α catalytic subunit. The variant of PTEN p.H123Y was present in one case (p13). These variants were sensitive to class I PI3K inhibitor. We also found PIK3C2B p.E545K variant in one case (p9), and PIK3C2G p.M1047I variant in another case (p3), both variants belong to class II PI3K. In addition, gene mutations were observed in *CDKN2A* in two cases (p18 and p21), *NF1* in two cases (p6 and p20), *DDR2* in two cases (p8 and p13), *PBRM1* in two cases (p9 and p13), *WHSC1L1* in one case (p10), *IRF4* in one case (p16), and *HRAS* in one case (p21).

The rearrangement *of anaplastic lymphoma kinase (ALK), c-ros oncogene 1, receptor tyrosine kinase* (*ROS1*), and *ret proto-oncogene* (*RET*) play a role on driving the occurrence and development of NSCLC (Takeuchi et al., 2012; Cancer Genome Atlas Research, 2014). These gene translocations were detected by NGS based large-gene panel test and RNA amplification assays in the present study. However, none of *ALK, ROS1*, and *RET* rearrangements were detected in the enrolled patients with lung ASC (data not shown).

### Detection of Somatic Copy Number Alterations in Lung ASC

**Figure 2:**
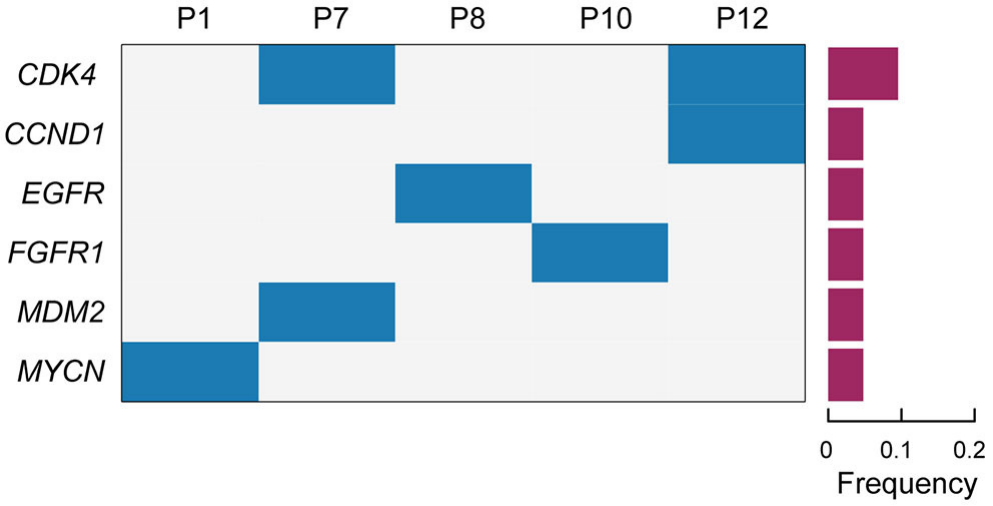
The profiles of somatic copy number alterations for the enrolled patients. Cases with somatic copy number alterations (CNA) are shown. Patient numbers are displayed in the upper lane. Gene names are listed on the left. The frequency of CNA are shown in a histogram on the right.

The somatic copy number alterations (CNA) play an important role in the development of lung cancer. In this study, CNA was detected in the enrolled patients with lung ASC. The results indicated that the amplification of six genes in tumor tissues were at least twice than in the normal tissues (Figure 2 and Table 2). Among those, the amplification of *cyclin dependent kinase 4* (*CDK4*) was observed in two cases (p7 and p12). Meanwhile, the coexisting amplification of *cyclin D1* (*CCND1*) and *CDK4* were detected in one case (p12). CDK4 and CCND1 are very important components involved in regulating the progress of G1/S phase in cell cycle. The complex of CKD4 and CCND1 has been studied as a therapeutic target for cancer (Malumbres and Barbacid, 2009; Musgrove et al., 2011). In addition, the tyrosine kinase receptors *EGFR* and *FGFR1* were amplified for eleven times in p8 and p10 cases, respectively. The present study also indicated p1 and p7 cases harboring *MDM2* and *MYCN* with copy number gains, respectively. Overexpression or amplification of *MDM2* occurs in a variety of cancer types.

**Table 2:** Copy number alterations of enrolled patients detected by targeted NGS panel.

**Patient ID**	**CCND1**	**CDK4**	**EGFR**	**FGFR1**	**MDM2**	**MYCN**
P1						6.1
P7		6.9			6.1	
P8			11.6			
P10				11.9		
P12	6.9	4				

### Association of Tumor Mutations Burden With Clinicopathological Characteristics

**Figure 3:**
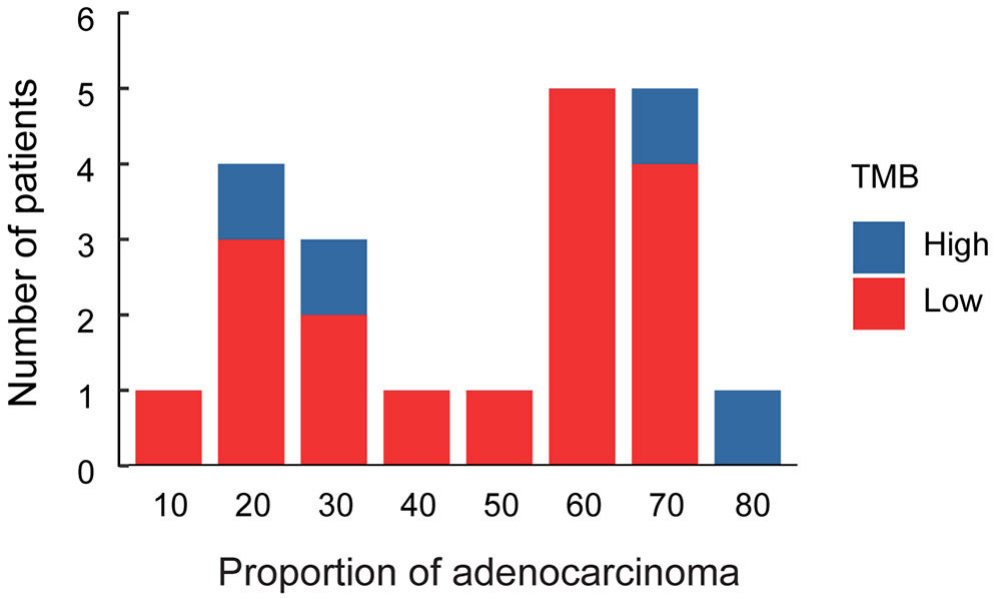
Histogram of proportion of adenocarcinoma cells in lung ASC. Red column represents patients with the low level of TMB, blue column represents patients with the high level of TMB.

Immune checkpoint inhibitor (ICI) therapies have earned its spurs in the treatment of malignant tumors in recent years (Gandhi et al., 2018). Tumor mutation burden (TMB) is a promising marker to predict survival after immunotherapy across multiple cancer types (Samstein et al., 2019). In our study, TMB value was determined in the enrolled patients. The results indicated that the median TMB was 5.25 mutations per megabases, with a range from 0.88 to 16.64 (Figure 1). We analyzed the association between TMB value and the proportion of adeno and squamous cells carcinoma of ASC (Figure 3). The results indicated that the high level of TMB was not significantly related to the high proportion of squamous cells in ASC of the lung. Meanwhile, the relationships between TMB level and the clinicopathologic features of adenosquamous cell carcinoma of the lung were analyzed. The results demonstrated that TMB value correlated significantly with pathological stages (*p* = 0.03) and lymph node (*p* = 0.03). The higher TMB value (cutoff ≥ 10 mut/Mb) was related to invasion of lymph node, while the lower TMB value (cutoff < 10 mut/Mb) was related to none of invasion of lymph node. However, there was no significant relationship between TMB and other clinicopathologic indexes, for instances, age, sex, smoking history, as well as *TP53* and *EGFR* mutations in lung ASC (Table 3). It was no distant metastasis in the enrolled patients. Therefore, we did not analyze the relationship between TMB level and the index of distant metastasis.

**Table 3:** Relationship between TMB index and clinicopathological characteristics of lung ASC.

**Characteristics**	**Numbers of patients (percentage)**	**TMB (cutoff** **=** **10)**		***p* value**
		**Low**	**High**	
Age				0.58
<70	15 (71.4%)	11	4	
≥ 70	6 (28.6%)	4	2	
Sex				0.63
Male	11 (52.4%)	8	3	
Female	10 (47.6%)	7	3	
Smoking history				0.30
Smokers	7 (33.3%)	4	3	
Non-smokers	14 (66.7%)	11	3	
Pathological stage				0.03
I-II	12 (57.1%)	11	1	
III	9 (42.9%)	4	5	
Tumor size (cm)				0.31
≤ 3	7 (33.3%)	6	1	
> 3	14 (66.7%)	9	5	
Lymph node				0.03
N0	12 (57.1%)	11	1	
N1-N2	9 (42.9%)	4	5	
TP53 mutation				0.59
w/t	8 (38.1%)	6	2	
With	13 (61.9%)	9	4	
EGFR mutation				0.27
w/t	11 (52.4%)	9	2	
With	10 (47.6%)	6	4	

### Relationships Between TMB and Clinical Outcomes

**Figure 4:**
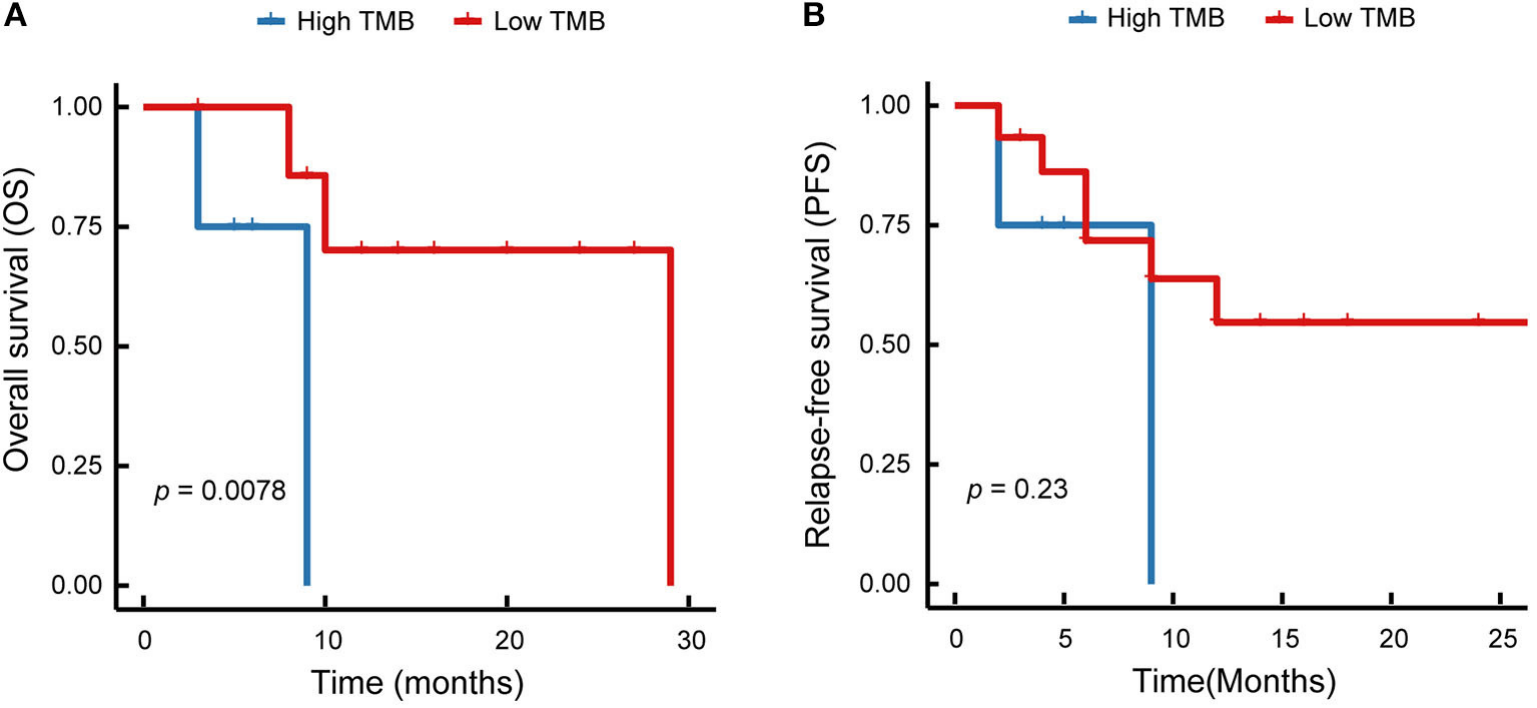
Kaplan–Meier survival curves of patients with lung ASC. **(A)** Overall survival of lung ASCs with high TMB and low TMB are shown. **(B)** Relapse-free survival of lung ASCs with high TMB and low TMB are displayed. Red solid line represents patients with low TMB, blue solid line represents patients with high TMB.

In this study, the enrolled patients underwent operation to completely resect the primary tumor tissues and accomplish the lymphadenectomy. In total, five (23.8 %) of 21 patients did not occur disease recurrence, 14 (66.7%) of 21 patients had local recurrence or distant metastasis, two (9.5) of 21 patients were out of contact. The Kaplan–Meier survival curves of relapse-free survival, and overall survival were displayed in Figure 4, with the median of 6 and 10 months, respectively. Among the enrolled patients, one case (p8) harboring EGFR exon 19del and amplification of EGFR, received the EGFR-TKI therapy. The patient developed brain metastases after 14 months of treatment with gefitinib. None of the enrolled patients treated with immune checkpoint inhibitor therapies.

We analyzed the relationship between TMB and survival time. The results demonstrated that TMB value was significantly correlated with the overall survival (*p* = 0.0078, Figure 4A), but not with the relapse-free survival (*p* = 0.23, Figure 4B). As shown in Figure 4, the high level of TMB was related to the short survival time. Therefore, immunotherapy might be a promising treatment option to improve the outcomes in lung ASC patients with high TMB.

## Discussion

The poor prognosis and fewer treatment option is still a clinical challenge for lung ASC. So far, a few studies introduced the mutational profile of lung ASC (Sasaki et al., 2007; Tochigi et al., 2011; Morodomi et al., 2015; Vassella et al., 2015; Shi et al., 2016; Lin et al., 2020), while most analyses were restricted to small gene panels. The continued studies are required to investigate genetic alterations and explore the potential therapies for lung ASC. The present study displayed the comprehensive analyses of somatic variations in lung ASC. In addition, it is the first study to reveal the clinical relevance of TMB level and PD-L1 expression in lung ASC.

Our study showed a high frequency of *EGFR* mutations in lung ASC, the mutation rate was 48%. However, in contrast to our observation, a lower prevalence of *EGFR* mutations was reported in lung ASC of Caucasian ethnic group, with a mutation rate of 13% (Tochigi et al., 2011). That might be due to the ethnicity differences between Asians and Caucasians. Moreover, consistent with the incidence of *EGFR* mutation in lung adenocarcinoma, the mutation rate was 46.7% in the Asian population and 15% in the white population (Liu et al., 2017). Furthermore, the current study revealed that the landscape of somatic variations of ASC was similar to that of lung adenocarcinomas, and supported the hypothesis that adenocarcinoma components and squamous cell carcinoma components of ASC shared a monoclonal origin (Lin et al., 2020). Therefore, considering the similar profile of somatic variations in ASC and lung adenocarcinoma, TKI might be an effective targeted agents for lung ASC with EGFR mutations. In our study, one resectable patient (pT2aN2M0) received EGFR-TKI therapy after four cycles of adjuvant chemotherapy, and had a clinical benefit from the treatment of gefitinib, with progress-free survival (PFS) of 14 months. In line with the observation, a current multicenter retrospective study also indicated that EGFR-TKIs were effective for patients with advanced ASC of lung, with the median PSF being 10.1 months (Lin et al., 2020). None of *ALK, ROS1*, and *RET* rearrangements were detected in our study. In line with our results, the previous study also did not find gene rearrangements in Caucasian patients with lung ASC (Vassella et al., 2015). That might be due to the lower prevalence of gene translocations in lung cancers and the small size of enrolled patients with lung ASC.

In addition, ICI therapies have been applied in treatment of malignant tumors in recent years. We valuated PD-L1 expression in tumor cells of the enrolled patients, while there were no significant associations between PD-L1 expression and the clinicopathologic features of lung ASC (Supplemental Table 4). Besides of PD-L1, TMB is a promising marker to predict clinical outcomes of patients with NSCLC to immunotherapy (Carbone et al., 2017; Hellmann et al., 2018; Samstein et al., 2019). The previous studies indicated lung squamous cell carcinoma harboring higher TMB than other solid cancer types (Vogelstein et al., 2013; Zhang et al., 2019). However, our results indicated that the high level of TMB was not significantly related to the high proportion of squamous cells in lung ASC. The current study displayed that TMB was lower in adenocarcinoma component than in squamous cell carcinoma component, with the median of 6.5 and 7.2 mutations/Mb, respectively (Lin et al., 2020). However, it is difficult to distinguish such small differences of TMB value in adenocarcinoma component and squamous cell carcinoma component.

In the present study, we also evaluated the relationships between TMB level and the clinicopathologic features and outcomes of lung ASC, though none of enrolled patients received ICI therapy. Our results indicated that the high level of TMB was related to the invasion of lymph node and the short survival time. Patients with the short survival time might be due to the invasion of lymph node. In line with the results, the previous study indicated that high TMB is a poor prognostic factor for the advanced NSCLC, as well as patient in early stage (Owada-Ozaki et al., 2018). However, as the limited numbers of enrolled patients, we did not obtain lung ASC with high TMB in early stage.

In conclusion, the lung ASC with high TMB might be associated with invasion of lymph node and short overall survival. Therefore, immunotherapy might be a potential treatment option for lung ASC patients with the high level of TMB.

## Data Availability Statement

The datasets presented in this study can be found in online repositories. The accession number is CNP0001586, the link is followed as: https://db.cngb.org/search/project/CNP0001586/.

## Ethics Statement

The studies involving human participants were reviewed and approved by Ethics Committee of Medical Research, the Second Affiliated Hospital of Nanchang University. The patients/participants provided their written informed consent to participate in this study.

## Author Contributions

XY and LW designed the project. YZ, YY, KL and YC performed the work. YZ wrote the paper. JW, BY, LX and CO-Y reviewed the paper. All authors accepted the final version of this manuscript for publication.

## Conflict of Interest

YY, LW, and YZ are currently employed by Berry Oncology Corporation. The remaining authors declare that the research was conducted in the absence of any commercial or financial relationships that could be construed as a potential conflict of interest.
